# Electron crystallography and dedicated electron-diffraction instrumentation

**DOI:** 10.1107/S2056989023003109

**Published:** 2023-04-14

**Authors:** Petra Simoncic, Eva Romeijn, Eric Hovestreydt, Gunther Steinfeld, Gustavo Santiso-Quiñones, Johannes Merkelbach

**Affiliations:** a Eldico Scientific AG, PARK INNOVAARE: delivery LAB, Villigen, Aargau5234, Switzerland; University of Kentucky, USA

**Keywords:** 3D electron diffraction, nano crystallography, instrumentation, electron diffractometer, 3D electron diffraction, nano crystallography, instrumentation, electron diffractometer

## Abstract

Electron diffraction (known also as ED, 3D ED or microED) is gaining momentum in science and industry. The application of electron diffraction in performing nano-crystallography on crystals smaller than 1 µm is a disruptive technology that is opening up fascinating new perspectives for a wide variety of compounds required in the fields of chemical, pharmaceutical and advanced materials research. Electron diffraction enables the characterization of solid compounds complementary to neutron, powder X-ray and single-crystal X-ray diffraction, as it has the unique capability to measure nanometre-sized crystals. The recent introduction of dedicated instrumentation to perform ED experiments is a key aspect of the continued growth and success of this technology. In addition to the ultra-high-speed hybrid-pixel detectors enabling ED data collection in continuous rotation mode, a high-precision goniometer and horizontal layout have been determined as essential features of an electron diffractometer, both of which are embodied in the Eldico ED-1. Four examples of data collected on an Eldico ED-1 are showcased to demonstrate the potential and advantages of a dedicated electron diffractometer, covering selected applications and challenges of electron diffraction: (i) multiple reciprocal lattices, (ii) absolute structure of a chiral compound, and (iii) *R*-values achieved by kinematic refinement comparable to X-ray data.

## Abstract 2

Recent years have seen a flurry of research activity in the field of electron diffraction. The introduction of the electron diffractometer, designed to be fully dedicated to its task of providing the best data from electron-diffraction experiments, will be a crucial factor for the continued growth and success of this technology.

## Introduction

With scientists in mind who don’t yet have an in-depth knowledge of 3D electron diffraction (3D ED) but a general understanding of X-ray diffraction methods, this article aims to provide an overview of the most important techniques, achievements, advantages, and challenges in the field. As the analysis of crystal structures of new materials is as important as ever, there is the need from industry and academia to determine crystal structures of smaller crystal sizes than previously possible. With experiments generally conducted on transmission electron microscopes, electron diffraction (ED) brings forth the ability to measure crystals in the range of tens to hundreds of nanometres in both direct (imaging mode) and reciprocal space (diffraction mode). The development of novel electron-diffraction techniques is therefore a good complement to established X-ray diffraction (XRD) methods in cases where crystals larger than 1 µm cannot be obtained. The boundaries of what it is possible to measure are being expanded as ED allows for the refinement of single-crystal structures that were previously too complex or too small for X-ray diffraction methods. Besides a comparison between X-ray diffraction and electron diffraction, special emphasis is given to dedicated 3D ED instrumentation and their requirements, as well as presenting benchmark experiments using such electron diffractometers.

### Differences between X-ray diffraction, neutron diffraction and electron diffraction

While the focus of this paper is electron diffraction (ED), many readers are likely more familiar with the principle of X-ray diffraction methods. Therefore, the authors consider it a helpful introduction to touch upon X-ray diffraction, and to a minor extent neutron diffraction, in order to highlight the ways that ED is unique – both its strengths and weaknesses. While all three rely on the diffraction of waves to probe crystal structures, yielding the basic unit-cell parameters and positions of the atoms, they differ in detail.

One of the key factors that determine the viability of a chosen method of single-crystal analysis is the sample size: a neutron beam requires the largest crystal size, typically greater than 1 mm^3^ is needed for single-crystal neutron diffraction (Koetzle & McIntyre, 2012 ▸), X-rays require crystals ranging from tens of microns (µm, micrometre) on in-house diffractometers, down to around 1 µm using synchrotron facilities. In contrast, crystals smaller than 1 µm can easily be measured by electron diffraction (Gemmi & Lanza, 2019 ▸).

Whereas electron beams and X-rays primarily interact with the electron clouds and nuclei of atoms (although in very different manners – see below), neutron beams primarily interact with the nuclei but also with unpaired electron spins. The irregular neutron cross section (*i.e*. the likelihood of interaction between a neutron and a target nucleus) allows neutron diffraction to easily identify light elements, such as hydrogen, even in the presence of heavy atoms (Blakeley *et al.* 2015 ▸). However, strong scattering may lead to high backgrounds, hindering structure determination.

Simplistically, as the name makes clear, X-ray diffraction uses an X-ray beam on a rotating crystalline sample and measures the angles and intensities of the diffracted beams. Since X-rays are waves of electromagnetic radiation, the atoms in a crystalline material can scatter the X-rays *via* the electrons of the atoms. The X-rays striking the electrons therefore produce secondary (spherical) waves, which emanate from the electron. This process is called ‘elastic scattering’ where electrons act as the scatterer. The waves cancel one another *via* destructive interference, except in reflection positions. In combination with an initial phase estimate, the intensities of these diffraction maxima allow the calculation of a 3D representation of the electron density within that crystal, which in turn gives the positions of atoms in the crystal structure. From this, we can determine the chemical bond lengths as well as other structural information. Since it is electron density that is probed by X-ray diffraction, light atoms are more difficult to localize by X-ray diffraction, especially in the presence of heavy atoms due to the effect of the diffraction pattern being stronger for heavier elements, as they have more electrons than light elements. Hydrogen, for example, having only one electron, is more difficult to detect with X-ray diffraction methods than heavier elements. Bonds to hydrogen generally appear too short because of the relatively high electron density between the atoms.

In electron diffraction, the same basic principle of scattering of a wave applies, but an electron beam is used. X-rays interact with the electron cloud, while in ED there is a Coulomb interaction of the electrons being scattered by the positive potential inside the electron cloud. Another difference is, since the wavelength of electrons of sufficient energy is much shorter than that of X-rays, even with the use of a synchrotron (0.025 Å *vs* 0.5–2.5 Å, respectively), the radius of the Ewald sphere is much larger for ED. As a result of the use of thin crystals in ED experiments, reciprocal lattice points become elongated and diffraction occurs even if the Bragg condition is not exactly satisfied, resulting in excitation errors (Williams & Carter, 1996 ▸), *i.e*. the deviation of the diffraction beam from the exact Bragg condition. As a consequence of the short wavelength, diffraction angles used in ED are also much smaller than XRD: 0 < 2Θ < 2° for ED *versus* 0 < 2Θ < 180° for XRD.

Since electrons interact more strongly with matter than X-rays (Henderson, 1995 ▸), sample thickness is the limiting factor for electron diffraction, *i.e*. even if the overall crystal is larger, sample thickness in the transmission direction cannot be more than 1 µm, ideally 500 nm or less (Martynowycz *et al.* 2021 ▸). According to Grüne *et al.* (2014 ▸), there is virtually ‘no lower size limit to the crystal’, with various studies successfully measuring sub-micron samples (*e.g*. Zuo *et al.*, 2003 ▸; Kolb *et al.*, 2011 ▸; Martynowycz *et al.* 2021 ▸). Grüne & Mugnaioli (2021 ▸) go on to point out that this ability to measure small crystal sizes less than 1 µm ‘aims to close the gap’ to the limitations of XRD, and indeed single micro crystals from powders down to around 10 nm can be analysed by ED. Furthermore, ED enables characterization of commercially important materials such as MOFs, zeolites, and pharmaceutical materials (*e.g*. Gemmi & Lanza, 2019 ▸; Mu *et al.* 2021 ▸), which are often intrinsically of small crystal size and cannot be grown to a suitable size for SC-XRD analysis.

While PXRD can be used for analysing small crystal sizes, peak overlap is often a concern in crystals with long cell parameters or because of phase impurities. However, with ED, single crystals can easily be isolated by TEM or STEM imaging and measured in a polyphasic mixture (Gemmi *et al.*, 2019 ▸). In addition, when synthesizing under hydrothermal conditions, it is not unusual to obtain multi-phasic powders with an average crystal size less than one micron. Zeolites or metal–organic frameworks often fall under this category, with highly disordered and complex structures – but can be solvable by ED (Steciuk *et al.*, 2021 ▸). Furthermore, drug development in pharmaceutical companies would potentially become significantly more efficient if there were no need to grow large crystals for single crystal X-ray diffraction (Bruhn *et al.*, 2021 ▸, Halford, 2022 ▸).

On the other hand, since single crystals are being analysed, minor impurities in a bulk sample might go undetected unless a statistically meaningful number of crystals are analysed. Powder XRD would be a faster method to determine the impurities in this case, but only if the sample and the impurity were available in sufficient quantities. Lacking a substantial amount of sample or impurity concentration, ED is more advantageous in this case. Automated high-throughput methods currently being developed will greatly contribute to handling phase mixtures or impurity characterization by electron diffraction (Wang *et al.*, 2019 ▸; Luo *et al.*, 2023 ▸).

### Challenges

As in all sciences, there is no one perfect solution to all the problems in crystallography. While electron diffraction offers many advantages and solutions to crystal-structure determination that other techniques do not, there are of course drawbacks and challenges.

#### High vacuum

As electrons are absorbed by air, electron-diffraction measurements have to be conducted under high vacuum, at pressures of 10^−6^ bar or lower. This is potentially problematic for hydrated or biological samples as they easily deteriorate under high vacuum. There are several methods to tackle this challenge: (i) Plunge cooling is used to stabilize the hydrated compounds by rapidly submerging the sample prepared on a TEM grid into cryogenic liquid, forming a thin layer of vitreous ice on the sample, and transferring it to the sample holder maintaining the low temperature (Dobro *et al.*, 2010 ▸). (ii) Vacuum-sensitive samples can be coated with sugar or low-pressure liquids such as ionic liquids (Tsuda *et al.*, 2018 ▸), (iii) *in situ* liquid cells can be used where electron-diffraction data is measured under ambient conditions (Karakulina *et al.*, 2018 ▸), and (iv) the crystals can be thinned using a focused ion beam (FIB) milling after embedding the sample into a solidifying material (Zhou *et al.*, 2019 ▸).

#### Radiation sensitivity

The first researchers utilizing 3D ED focused on more robust, inorganic materials that did not easily suffer from beam damage. However, with a continuous method of data collection, a faster and lower beam dose became possible, paving the way for more sensitive materials to be analysed. Materials that are sensitive to beam damage often pose a limiting factor for analysis, which cooling with liquid N_2_ or He can help circumvent. This cryo-fixation is often the preferred method to stabilize sensitive materials such as hydrated or biological samples that would otherwise be damaged under vacuum (Andrusenko & Gemmi, 2022 ▸). As demineralized water does not evaporate significantly at 100 K, it is the preferred cryoprotective agent to cover the sample with a vitreous layer of ice. However, the interaction of the cryogenic liquid and the sample needs to be taken into account, as some powders will dissolve in water. A downside to cryo-plunging is that possible artefacts during sample preparation or electron- beam interaction may be present, as well as the need for the use of specific cryo-equipment.

A detailed methodology and discussion of acquiring high-resolution data on highly sensitive 2D biological crystals using cryogenic methods can be found in Gonen (2013 ▸) and Bruhn *et al.* (2021 ▸).

#### Dynamical scattering

The very phenomenon that makes ED so powerful for studying nanometre-sized samples, *i.e*. the strong interaction of the electrons and matter, can also lead to one of the largest problems: dynamical scattering. In contrast to the interaction between X-rays and matter, the assumption of single, or kinematic, scattering is not true for electron diffraction. Rather multiple or dynamical scattering occurs, *i.e*. the scattering of different beams occurs multiple times within a sample. As a consequence for structure elucidation, the linear relationship between the reflection intensity and the square modulus of the structure factor breaks down. Dynamical scattering effects were especially prominent in electron-diffraction data collection along a zone-axis, which was the common mode for collecting data in the early years of electron diffraction. As electron diffraction became more established as a structure-identification technique, various data-collection protocols arose to circumvent the problem of dynamical scattering (refer to Section 1.3 for more details). All these methods have the underlying solution that diffraction patterns need to be collected using a series of tilt or rotation runs on a randomly oriented crystal (Gemmi & Lanza, 2019 ▸), which reduces dynamical scattering effects. However, kinematic refinements of ED data still suffer from high *R*-values and less accurate structure models compared to X-ray diffraction data. By applying dynamic scattering theory, data processing now allows for greatly improved structure refinement (Petricek *et al.*, 2014 ▸), yielding excellent results with increasing accuracy of atom positions and bond lengths, correct light- and neighbouring atom positions and determination of absolute configuration (Palatinus *et al.*, 2015*b* ▸).

### Overview of electron-diffraction data-collection methods

**Figure 1:**
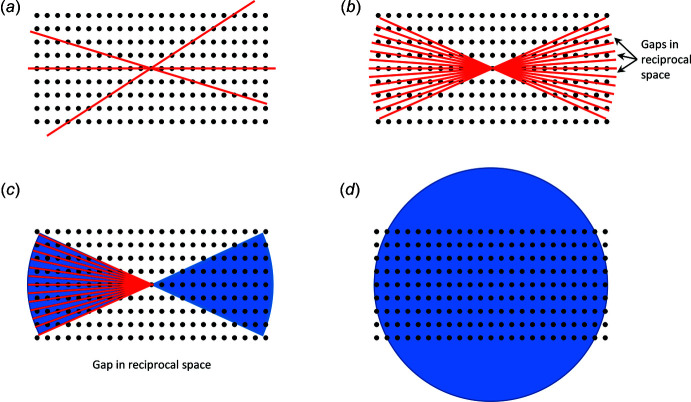
(*a*) ED data collection along zone axes (high-symmetry orientations of the crystal), (*b*) ED data collection in fixed tilt steps, (*c*) left: PEDT and RED data collection (simplified representation); both protocols attempt to fill the reciprocal space between the tilt steps, right: cRED data collection (simplified representation), (*d*) under ideal conditions, 360° continuous rotation on ED-1 allowing a complete sampling of reciprocal space.

The intrinsic challenges of electron diffraction such as dynamical scattering and excitation errors are also reflected in the development of ED data-collection methods, as outlined in the following historical overview. The discovery of electron diffraction goes back to the experiment conducted by Davisson and Germer in 1927 (Davisson & Germer, 1927*a* ▸,*b* ▸) showing the scattering of electrons from a nickel crystal and confirming the wave nature of electrons predicted by de Broglie. ‘For their experimental discovery of the diffraction of electrons by crystals’, Davisson and Thomson were awarded the Nobel prize in 1937. In the post-war years, electron diffraction turned into a niche technique in comparison to the method of choice for structure elucidation, single-crystal X-ray diffraction. Yet, during the 1950s and 1960s, Vainshtein and co-workers performed pioneering and fundamental research on electron diffraction (Vainshtein, 1964 ▸) encompassing the calculation of atomic scattering factors of electrons for all chemical elements, the use of electrostatic potential for the localization of atoms, and the determination of various organic and inorganic structures using electron diffraction (Klechkovskaya & Imamov, 2001 ▸). In the 1990s, Dorset (1995 ▸, 1996 ▸) and Weirich *et al.* (1996 ▸) revived the field of research and showed the crystallographic community that incredibly small crystals of tens to hundreds of nanometres in size could be efficiently characterized using electron diffraction on a TEM. However, refinements were still troublesome because of intrinsic challenges such as dynamical scattering and excitation error effects, resulting in time-consuming and awkward structural-determination procedures, as data were collected while the crystals were aligned along a crystallographic zone axis [*i.e*. a high symmetry orientation of the crystal, see Fig. 1 ▸(*a*)], as outlined in more detail in Section 1.2 and also summarized by Gemmi *et al.* (2019 ▸) and Saha *et al.* (2022 ▸). Later, Kolb and her colleagues (Kolb *et al.*, 2007 ▸) determined that the best method was instead to collect electron-diffraction data off-axis (not pre-oriented) in a series of rotational steps that can then be combined to recreate the 3D information, known as automated diffraction tomography, or ADT.

Since then, various techniques and protocols have been developed to handle the data acquisition of crystal structures through electron diffraction. These include rotation electron diffraction (RED), continuous rotation electron diffraction (cRED), electron-diffraction tomography (EDT), integrated electron-diffraction tomography (IEDT), precession electron-diffraction tomography (PEDT), and microcrystal electron diffraction (microED). However, Gemmi *et al.* (2019 ▸) proposed combining all of these acronyms to summarize the general methodology: 3D Electron Diffraction (3D ED). They conclude that this is a generic term that can be used when describing the method of tilting a sample around an arbitrary axis and sampling the whole 3D reciprocal space using ED.

The resolution of such nanostructures has increased thanks to two major developments:

As the possibilities and advantages of using ED, both alone or alongside XRD, become more apparent, the number of research groups utilizing ED in turn grows. Indeed, a simple internet search for ‘elusive structure + 3D Electron Diffraction’ will yield an ever-increasing number of journal articles across a variety of research fields where, thanks to ED, such structures have finally been resolved. These range from natural minerals that can potentially help with carbon capture (*e.g*. Krysiak *et al.* 2021 ▸), to common pharmaceutical ingredients that have not been fully analysed (*e.g*. Wang *et al.* 2017 ▸). In a certainly incomplete overview, some of the more established groups that focus on ED across a variety of fields are summarized below:

Focusing on structural biology, Tamir Gonen’s group at the University of California developed a technique they named MicroED, based on CryoEM methods and allowing crystallographic refinement to 2.5 Å resolution (Nannenga *et al.* 2014 ▸; Hattne *et al.* 2015 ▸). In 2021 the group found that macromolecules could be refined down to sub-ångstrom resolution (Clabbers *et al.*, 2022 ▸).

Mauro Gemmi, the Principal Investigator for Electron Crystallography at the Instituto Italiano di Tecnologia in Pisa, was one of the first researchers to utilize precession electron diffraction to solve crystal structures. His group coined the phrase ‘3D ED’ to summarize the techniques that used 3D reciprocal space electron-diffraction experiments (Gemmi *et al.*, 2019 ▸). His current research is focused on analysing beam-sensitive materials such as organics, hybrid crystals and proteins using low-dose 3D ED techniques, one goal being to show that ED can be effectively used across a spectrum of research fields in crystallography for organic and inorganic materials. Most recently, this has included nanocrystals in the pharmaceutical industry (Andrusenko & Gemmi, 2022 ▸), mineralogical applications (Mugnaioli *et al.*, 2022 ▸; Toso *et al.*, 2022 ▸), medical research (Del Turco *et al.*, 2022 ▸) and material science (Kleain *et al.*, 2020 ▸; Hamon *et al.*, 2022 ▸).

Tim Grüne heads the Centre for Chemical Structure Analysis, University of Vienna. While his laboratory also utilizes X-ray diffraction for structural analysis, the bulk of his publications and research are in the ED field. He is a strong promoter of ED, believing that electron crystallography should be a ‘common part of the analytical chemistry toolkit’ (Grüne *et al.* 2021 ▸).

Under the lead of Xiaodong Zou, the electron crystallography and analytical TEM research group at the Stockholm University has developed several methods and software packages for structure determination using electron diffraction, in particular for automated data collection and processing (Cichocka *et al.*, 2018 ▸; Wang *et al.*, 2019 ▸; Roslova *et al.*, 2020 ▸). In addition, their research is focused on electron-crystallography studies of zeolites, metal–organic frameworks and proteins (Xu *et al.*, 2019 ▸; Zou, 2019 ▸; Ge *et al.*, 2021*a* ▸).

Ute Kolb’s research group at the Centre for High Resolution Electron Microscopy (EMC-M), Johannes Gutenberg University Mainz, Germany, has been working on developing a simple and reliable method for analysing nanocrystals since 1997. Her group pioneered the technique of collecting data off-axis, allowing for the minimization of dynamical effects (Kolb *et al.*, 2007 ▸).

At the Institute of Physics of the Czech Academy of Sciences, Lukas Palatinus and his group have spearheaded the development of theoretical concepts for electron-diffraction structure elucidation, investigating the use of dynamical scattering to determine the absolute structure of chiral compounds (Petricek *et al.*, 2014 ▸; Palatinus *et al.*, 2015*a* ▸,*b* ▸, 2017 ▸, 2019 ▸; Brázda *et al.*, 2019 ▸; Steciuk *et al.*, 2021 ▸).

Various methods and protocols for 3D electron diffraction have been developed, resulting in specific methods of data acquisition. All rose out of the need to work around the limitations of ED, namely to reduce dynamical effects, reduce data-collection times and beam exposure, and increase the number of collected reflections. All data-collection protocols have in common that the crystal is rotated around a random axis, commonly by stepwise tilt angles. There are currently several methods of data acquisition for 3D electron diffraction:

The precession electron diffraction (PED) method was first proposed by Vincent & Midgley (1994 ▸), in which a crystal is oriented along a zone axis and the beam is precessed on the sample surface along a conical path. This beam manipulation resulted in reflections from off-axis orientations. PED therefore greatly improved challenges due to dynamical scattering and excitation errors. However, data-collection protocols remained cumbersome as crystals had to be reoriented along other zone axes and datasets had to be merged in order to obtain acceptable data completeness.

The next big step was the automated diffraction tomography (ADT) method, by which data is collected from a homogenous sample in a stepwise manner with fixed tilt steps (Kolb *et al.*, 2007 ▸). The methodology uses a small tilt-step size, with a short exposure time [Fig. 1 ▸(*b*)]. This results in a large reciprocal space coverage, and by not exposing the sample to the beam between steps, reduces the amount of potential beam damage to the sample. The structure can be directly solved from the acquired data. However, this method has a large drawback of gaps in reciprocal space that remain unsampled due to the stepwise data collection (Saha *et al.*, 2022 ▸). To overcome this challenge, stepwise data acquisition can be complemented with a precession of the electron beam, a technique called PEDT [Fig. 1 ▸(*c*), left; Mugnaioli *et al.*, 2009 ▸; Kolb *et al.*, 2011 ▸].

Supported by custom software, rotation ED (RED) aims to fill in the gap between the relatively large rotational step, with minor tilt steps of approximately 0.1° in combination with electron beam tilts [Fig. 1 ▸(*c*), left; Zhang *et al.* 2010 ▸]. However, while this still results in gaps in data, it is still possible solve the crystal structure with direct methods (Gemmi *et al.*, 2015 ▸).

**Figure 2:**
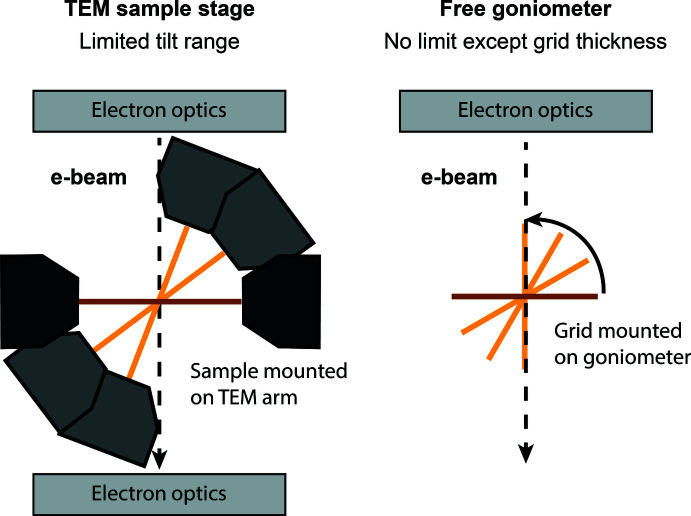
Illustration of limited tilt angle in conventional TEM (left) *vs* 360° rotation in the Eldico ED-1 (right).

The most recent development for data collection is a continuous method (cRED), using a goniometer rotating at an accurately controlled continuous speed [Fig. 1 ▸(*c*), right; Nederlof *et al.*, 2013 ▸; Nannenga *et al.*, 2014 ▸]. This utilizes a lower electron-beam dose, and is generally a faster method for ED data acquisition. The potential of this method is greatly enhanced by detectors with a fast readout time such as the complementary-metal-oxide-semiconductor (CMOS) detectors in rolling-shutter mode (Nannenga *et al.*, 2014 ▸) or hybrid-pixel detectors by preventing the ‘gaps’ in data that may occur in stepwise methods. However, large gaps still remain in the data collection because of the limited tilt angle of the TEM arm (see also Section 2, Fig. 2 ▸).

### Applications

#### Light elements

A good signal-to-noise ratio due to the higher relative scattering power of the lighter elements (Gemmi *et al.*, 2019 ▸) allows for light elements such as Li and H to be found, and using dynamical refinement of electron-diffraction data, light atoms can accurately be located. Hydrogen atoms can be determined in both organic and inorganic materials (Palatinus *et al.*, 2017 ▸). Even weak ordering of OH/H_2_O groups in the pores of zeolites could potentially be determined with ED using cryo-plunging protocols (Mugnaioli *et al.*, 2020 ▸). Since zeolites have a wide range of uses in various industries, it is important to fully characterize them. Many of the crystals that are synthesized under hydrothermal conditions consist of multiphasic powders with an average crystal size below one micron, making them difficult to fully characterize. In addition, substitutions in the framework, such as Li for Zn, could be missed using only synchrotron data. However, by being able to identify these light-element substitutions in small crystals, new phases may be resolved (Steciuk *et al.*, 2021 ▸).

#### Chirality/absolute structure

In pharmaceuticals, chirality and determining the absolute configuration of a molecule is essential – while one configuration of isomers can be beneficial, a contamination with a different isomer can have adverse effects. Normally, XRD is the method to determine the absolute structure of these molecules. However, there are many new compounds being developed for which it is too difficult to grow large crystals, resulting in the need for an alternative solution. This is where electron diffraction can play an important role (Brázda *et al.*, 2019 ▸). For example, Brázda and colleagues successfully analysed a highly unstable form of a pharmaceutical co-crystal of sofosbuvir and l-proline. Previously, analysis of such compounds was not possible due to instability of the crystalline structure under the available conditions, *i.e*. that materials would need to maintain their crystallinity after an electron fluence of at least one electron per square ångström. In the same study, he showed that materials composed of light scatterers could still have their absolute structure determined without any ambiguity.

#### Twinning and multiple lattices

Along with the obvious advantage of being able to measure extremely small crystal sizes, ED also is a very good solution when it comes to handling twinning, intergrown crystals, or solving and refining structures of multiple crystals from a single data collection. Uncertainty in the refinement procedure and space-group determination can be a common problem when confronted with crystallographic twins or aggregates. Light microscopy does not always reveal whether a crystal is single, especially if the multiple components are not easily distinguishable. Twinning, in particular, may only be revealed after extensive XRD measurements have been taken. While software exists to help with the XRD refinement of twins, there is no guarantee that they can be correctly resolved. For example, in merohedric twins, an incorrect higher symmetry space group is often assigned due to perfect overlap of the individual reciprocal lattices.

Where 3D ED shines in this regard is the combination of imaging and diffraction performed on the same crystal. Aggregates of crystals might appear under a light microscope, but single components can be identified using the imaging mode on a TEM. This enables collection of data from regions as small as the beam diameter, which greatly increases the likelihood of measuring only one individual at a time. Automated Diffraction Tomography (ADT) has been used for the structure elucidation of a bismuth–metal–organic framework (Feyand *et al.*, 2012 ▸). Another excellent example of this is the case of orthocetamol, a regioisomer of paracetamol. It has monoclinic symmetry that tends to twin on the scale of tens of nanometres. As it is a very promising compound in pharmaceutical development, it was essential to determine its crystal structure. As a result of its intricate twinning, it was deemed unsuitable for measurement with XRD. Thanks to the development of 3D ED methodologies, however, the structure was finally able to be resolved, including the location of several H atoms (Andrusenko *et al.*, 2019 ▸).

Further considerations of using electron diffraction to process multiple crystals in a single data collection will be discussed later in this paper, under instrumentation and case studies.

## Dedicated electron diffraction instrumentation

As outlined in the introductory section above, 3D ED has seen a tremendous increase in research activities by various groups over the last few years. Until now, most ED experiments have been performed on modified transmission electron microscopes (TEMs). The key characteristics of said instrumentation, covering electron sources, choice of energy, detectors, and corresponding experiments including sample preparation, working under vacuum, centring the crystal and data-collection routines have been reviewed by Grüne & Mugnaioli (2021 ▸). While the approach of using TEMs for 3D ED makes the technique accessible to many research groups, it comes with various disadvantages. As a result of the complex instrumentation, ED performed on TEMs remains a highly specialized technique for microscopy experts. The costly microscopy instruments are generally optimized for imaging, making the collection of electron-diffraction data cumbersome and time consuming. Furthermore, the instruments are not specifically aimed at crystallographers. As diffraction experiments are conducted on a TEM sample stage, sample manipulations in *x*, *y*, *z* and tilt are limited and do not have the benefits of a goniometer, as used in X-ray diffraction. Sample tracking during rotation data collection is often necessary as the stage can move out of the beam, making experiments challenging (Gemmi *et al.*, 2015 ▸; Lanza *et al.*, 2019 ▸). The lack of commercial hardware and software solutions has led many research groups to develop their own data-collection and software routines to collect ED data on TEMs for structure determination (Kolb *et al.*, 2007 ▸; Zhang *et al.*, 2010 ▸; Wan *et al.*, 2013 ▸; Nederlof *et al.*, 2013 ▸; Wang *et al.*, 2017 ▸). To achieve the widespread advantages of ED, dedicated ED instrumentation and software are key. Therefore, the introduction of dedicated ED hardware is of great importance. A key aspect of the recent boost in 3D ED-related research has been the development of ultra-high-speed hybrid-pixel detectors, as they enable fast data collection during ED rotation experiments because there is no dead time, noise-free electron counting and shutterless operation (Grüne & Mugnaioli, 2021 ▸). Hybrid-pixel detectors are available from several commercial providers (ASI Amsterdam Scientific Instruments, 2023 ▸; Fernandez-Perez *et al.*, 2021 ▸; Quantum Detectors, 2023 ▸; Rigaku Corporation, 2023 ▸; X-Spectrum, 2023 ▸) as well as the ‘Jungfrau’ detector developed at the PSI Switzerland (Paul Scherrer Institute, 2023 ▸; Mozzanica *et al.*, 2016 ▸). However, other hardware and software aspects in 3D ED research remain less optimized.

Heidler *et al.* (2019 ▸) have laid out the foundation of requirements for a dedicated and optimized electron diffractometer, (i) parallel beam at the sample, (ii) STEM imaging for low-dose crystal imaging, (iii) a high-precision goniometer, (iv) rapid access to experimental parameter, (v) energy filter, (vi) horizontal layout.

Several commercial approaches are currently available for electron diffraction, three of which are based on optimized TEMs.

ThermoFisher Scientific offers the combination of a cryo-TEM with a MicroED package and a scintillator-based camera, Ceta-D, optimized for low-dose diffraction data collection (ThermoFisher Scientific, 2019 ▸). The cryo-TEM provides a stable column for switching between imaging and diffraction modes, as well as a sample stage for continuous tilt. The MicroED package includes dedicated software for crystal and diffraction screening, and optimized optics settings and hardware for ED data collection.

In a collaboration with JEOL Ltd., Rigaku introduced an electron diffractometer, the XtaLAB Synergy-ED in 2021, covering both hardware and software improved for 3D ED (Ito *et al.* 2021 ▸). It features an integrated and ED-optimized software workflow based on Rigaku’s *CrysAlis PRO*, and hardware from JEOL including a 200 kV electron source and optical system, an ED dedicated hybrid-pixel detector HyPix-ED by Rigaku, and an *x*, *y*, *z* and tilt sample stage to collect ED data by the continuous rotation method.

Tescan has recently introduced the Tensor, a 4D STEM system also offering precession electron diffraction capabilities (Tescan, 2022 ▸).

Nanomegas offers DigiSTAR, an advanced TEM electron-diffraction tool for nanocrystal structure determination to perform precession electron diffraction, which is compatible with most TEMs (Nanomegas, 2022 ▸).

While these commercial solutions contribute significantly to the continued success of 3D ED, they do not address two key points of an electron diffractometer highlighted by Heidler *et al.* (2019 ▸): (i) the high-precision goniometer, which is considered the most important hardware to be improved for an electron diffractometer, and (ii) the horizontal layout. Because of the small sample size and the small beam size in ED experiments, the stability and accuracy of the translation and rotation movement of the goniometer are crucial for high-quality data collection. The sphere of confusion of the goniometer needs to be as small as possible (< 500 nm) to ensure that the crystal does not move out of the beam during the rotation ED experiment.

Both features of a high-precision goniometer and a horizontal layout have been implemented in Eldico’s ED-1 having the following standard configuration: (i) a horizontal beam direction with a beam diameter of 40 nm in imaging mode and 200–1000 nm in diffraction mode, (ii) a 160 kV LaB_6_ electron source, (iii) a reduced set of lenses optimized for changing between imaging and ED modes at the click of a button, (iv) a bright-field STEM detector, (v) a five-axis translational goniometer with 360° φ rotation and a vertical goniometer axis, (vi) a large sample chamber of more than 12 cm in diameter, (vii) a hybrid-pixel ‘Quadro’ detector from Dectris and (viii) software for instrument control and data collection (Eldico Scientific AG, 2022*a* ▸). Depending on the experimental setup, the angle of φ rotation might be reduced ±100° for a low-temperature goniometer configuration because of the coolant lines attached to the goniometer.

**Figure 3:**
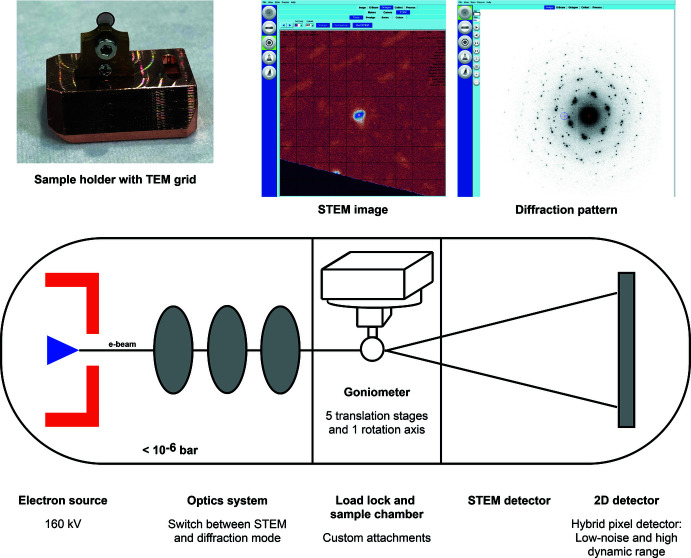
Eldico ED-1 electron diffractometer. Top row: Sample holder with TEM grid to fit on goniometer; STEM image and diffraction pattern, visualizing switching between imaging and diffraction modes. Bottom row: Principle horizontal layout showing key components.

The horizontal layout and vertical goniometer axis results in an instrument design familiar to X-ray crystallographers, enabling the benefits highlighted by Heidler *et al.* (2019 ▸). The ED-1 features multiple magnetic lenses with variable focal lengths and a single beam-limiting aperture with a fixed aperture diameter between the electron source and the sample, designed *ab initio* and fully optimized for the most accurate diffraction experiments, as well as for imaging in order to visually inspect and evaluate the sample (Niebel *et al.* 2021 ▸), but without optics after the sample, resulting in reduced distortion of the diffraction pattern as there is no objective nor intermediate lens in the ED-1 (Brázda *et al.*, 2022 ▸). The STEM detector allows low-dose imaging to be used for crystal location and centering, reducing radiation damage on the sample. The goniometer is the technical centrepiece of ED-1 enabling the selection and alignment of any crystal on a sample grid, the high accuracy of the five degrees-of-freedom results in a small sphere of confusion, keeping the crystal in the beam for the full rotation, while high-speed motors reduce the accumulated dose during sample alignment, evaluation and measurement. Crystal tracking (Kolb *et al.*, 2011 ▸; Lanza *et al.*, 2019 ▸; Yang *et al.*, 2022 ▸) during a rotation measurement is no longer necessary, resulting in even more reduced radiation exposure. As there are no lenses present downstream of the sample, a spacious sample environment can be realized, allowing the implementation of a five-axis goniometer and access to various custom attachments for cryo-ED or *in situ* studies. The Eldico ED-1 is equipped with a Dectris Quadro detector (Fernandez-Perez *et al.*, 2021 ▸) with the following specifications: (i) zero-noise read-out, (ii) single-electron sensitivity with a detective quantum efficiency DQE > 0.8, (iii) full frame rate of 2250 (16-bit) or 4500 (8-bit) fps. The software enables intuitive switching between pre-set imaging and diffraction modes within seconds. Furthermore, the software allows highly flexible data export suitable for data processing by various (X-ray) crystallographic software packages such as *APEX* (Bruker, 2021 ▸), *DIALS* (Winter *et al.*, 2021 ▸), *XDS* (Kabsch, 2010 ▸) or *PETS* (Palatinus *et al.*, 2019 ▸). An overview of the design of the ED-1 diffractometer featuring the horizontal layout is shown in Fig. 3 ▸.

The optimized optical design and the use of a goniometer also allow a larger rotation angle as shown in Fig. 2 ▸. The rotation angle of a grid installed on a TEM arm is limited by the geometry and dimensions of said arm and the electron optics. While continuously rotating the TEM arm, either the outer edge of the arm will hit and block the beam from reaching the sample or the rotation range will be limited by the pole pieces of the electromagnetic lenses (depending on the TEM design). In contrast, a grid installed on a so-called free goniometer as installed on an ED-1 can rotate a full 360° [Fig. 1 ▸(*d*)]. In an actual experiment, the ideal full 360° rotation is potentially limited by (i) the grid thickness, (ii) the location of the crystal within a hole on the grid and (iii) the radiation sensitivity of the crystal (*i.e.* the crystal deteriorates during data acquisition). Therefore, a selection of the continuous rotation range of 100–180° is often made during ED data collection to obtain the best quality diffraction data. The TEM design also puts further limitations on the areas of the grid that can be properly centred in the electron beam, whereas the whole grid is accessible on a free goniometer.

The combination of bespoke specifications enables acquisition of high-quality electron diffraction data and completeness for structure elucidation for a wide range of samples, even for beam-sensitive compounds such as organic molecular crystals.

## Use cases on Eldico ED-1

The Eldico ED-1 diffractometer has been validated and benchmarked with the following four use cases, highlighting various types of known compounds and their crystallographic challenges. Sample preparation and diffraction experimental setup were common to all use cases.

Before diffraction experiments, it has to be ensured that samples have a thickness small enough to be measured by electron diffraction. The suitable thickness for the electrons to penetrate depends on the type of compound to be analysed. While organic crystals with a thickness of up to 1 µm can be measured, inorganic compounds need to be significantly thinner, ideally less than 100 nm. As a result of the small size of the electron beam, it is often possible to measure just at the edge of a crystal, which is likely thinner than the bulk of the crystal. The high-precision goniometer will ensure that even the edge of the crystal will remain in the beam while conducting the rotation experiment.

Samples were generally obtained from commercial sources (see CAS numbers in Table 2 ▸) and were prepared by grinding a small amount of the sample gently between two glass slides. The sample was scraped off the glass slide with a metal spatula, and the ground solid was deposited on a carbon-coated Cu grid, achieving good dispersion of microcrystals over the grid. The prepared grid was mounted on the sample holder and inserted into the diffractometer *via* a load lock to preserve the high vacuum inside the diffractometer.

Crystals with suitable size and thickness were identified by STEM imaging using a 5 mm diameter photodiode (Opto Diode, 2019 ▸). The diffraction quality of the selected crystals was assessed by taking a single-shot diffraction image. In case of suitable thickness and diffraction quality, the crystal was centred in *x*, *y* and *z* using a crystal alignment routine: (i) the crystal to be measured is visually centred in STEM mode, (ii) the crystal is gradually rotated around the ϕ-axis and automatically readjusted to a centred location. The instantaneous switching between STEM and diffraction modes enables an efficient crystal selection and centring procedure. After the centring of the selected crystal has been finalized, ϕ-scans using the continuous rotation method were performed. A ϕ-scan corresponds to an alpha tilt on transmission electron microscope.

### Electron diffraction of multiple crystals – tyrosine

This use case addresses the aspects of merging data sets in electron diffraction and dealing with crystal conglomerates on grids. Merging sets of data collections from rotation electron-diffraction experiments is a common practice. As a result of a reduced rotation range, the completeness of a single-rotation data collection might be limited. In order to improve the completeness of structure refinement, data sets from different ϕ-scans can be combined. While structure elucidation using single-crystal X-ray diffraction frequently has to deal with intergrown crystals (real twins), both twinning and multiple crystals in the electron beam can pose a challenge in electron-diffraction experiments. As a result of the peculiarities of sample preparation using TEM grids for electron-diffraction data collection, single crystals on the grid are often not isolated but occur in conglomerates. Therefore, the electron beam might hit several crystals during a single ϕ-scan, resulting in randomly oriented, multiple reciprocal lattices sampled in the same ϕ-scan. The ability to resolve diffraction patterns of multiple crystals during structure elucidation with data collected on Eldico ED-1 has been demonstrated on the amino acid tyrosine. In addition, higher completeness could be reached by combining two data sets collected in a single ϕ-scan (Eldico Scientific AG, 2022*a* ▸,*b* ▸,*c* ▸,*d* ▸).

**Figure 4:**
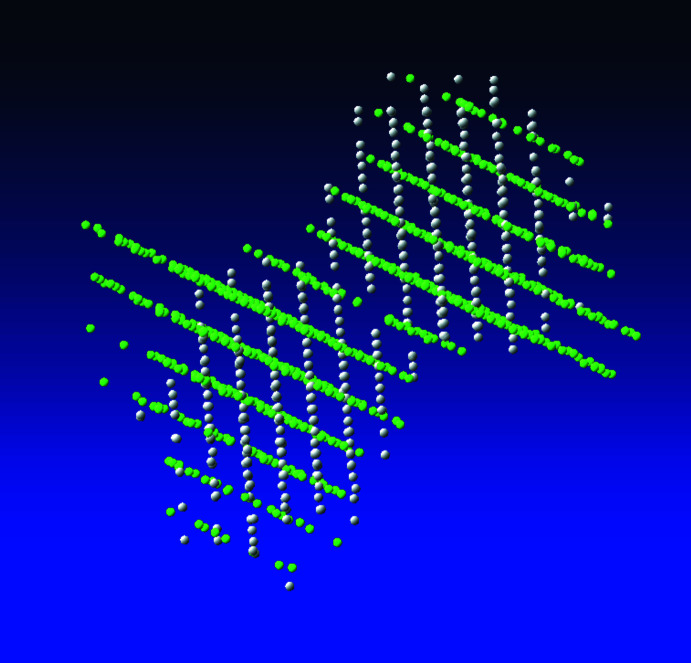
The reciprocal space visualization in *RLATT* to divide the reflections into two groups corresponding to the two crystal orientations.

Single-scan continuous rotation data were collected at 160 keV (0.02851 Å) at ambient temperature. The ϕ range was −70° to 30° at an increment of 1° with an exposure time of 0.5 s per frame. The total measurement time was 50 s. Data processing and handling of multiple reciprocal lattices was performed using the *APEX4* software package and implemented programs (Bruker, 2021 ▸). The reciprocal space visualization in *RLATT* was used to divide the reflections into two groups corresponding to the two crystals (Fig. 4 ▸). Indexing of these groups yielded the same unit cell (within standard deviations), but with different orientations (rotation by *ca* 47°). Both diffraction patterns were integrated and corrected for Lorentz and polarization effects, scan speed, background, and absorption using *SAINT* (Bruker, 2021 ▸) and *SADABS* (Krause *et al*., 2015 ▸) using an additional spherical absorption correction μ*r of 0.5, a semiempirical value chosen to reduce dynamical effects. Space-group determination based on systematic absences and *E* statistics, as well as data merging was performed in *XPREP* (Sheldrick, 2008 ▸). The structure was solved by *SHELXT* (Sheldrick, 2015*a* ▸) and refined with *SHELXL* in conjunction with *ShelXle* (Sheldrick, 2008 ▸; Hübschle *et al.*, 2011 ▸, Sheldrick, 2015*b* ▸) against all data using the mapping operation derived from the relation of the two crystals (Table 1 ▸). All hydrogen atoms could be located in the difference-Fourier map and were refined with a riding model with inter-nuclear distances (Grüne *et al.*, 2014 ▸) as opposed to ‘X-ray distances’.

**Figure 5:**
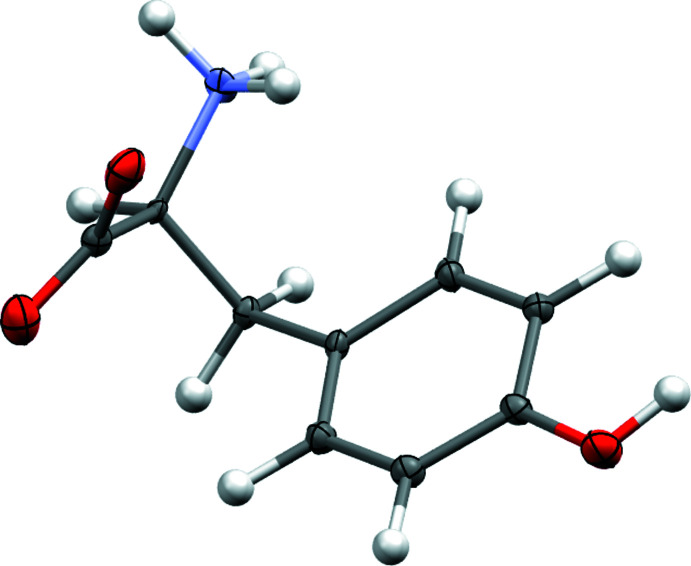
Molecular structure obtained from the merged data set for tyrosine. Generated using *Mercury* (Macrae *et al.*, 2020 ▸)

As discussed above, *R*-values of structure refinement from electron-diffraction data tend to be higher compared to single-crystal X-ray diffraction data. For this study, *R*-values of 14.05% (*R*_1_) and 37.68% (*wR*_2_) were obtained (Fig. 5 ▸). Considering the merging of a two-lattice structure, this is a promising outcome for electron-diffraction structure elucidation. As two crystals were measured in a single ϕ scan, the data sets for the two domains had a completeness of 80.4% and 59.9% and could subsequently be merged to obtain completeness of 86.7%. Crystal information and refinement statistics of the merged data set are summarized in Table 2 ▸.

### Absolute configuration of a chiral compound – histidine

Many crystalline compounds have Sohncke space groups and display chiral structures. Understanding the absolute configuration of chiral molecules is of great importance for the pharmaceutical industry as enantiomers can have dramatically different pharmacological properties. Therefore, it is crucial to know which enantiomer is the correct one, and whether the resulting sample of a chemical synthesis is enantiopure. According to Palatinus *et al.* (2015*a* ▸) and Brázda *et al.* (2019 ▸), the absolute structure can be determined from electron diffraction, because it takes into account the inversion symmetry violation due to multiple scattering. In order to determine the absolute structure, the collected data have to be accurate enough to clearly observe intensity differences between Friedel pairs. Even for organic compounds with only light elements, this is the case in ED. The correct enantiomer can then be determined by comparing the *R*-values of the original and inverted structure from the refinement (Palatinus *et al.*, 2015*b* ▸; Brázda *et al.*, 2019 ▸).

Single-scan continuous rotation data were collected at 160 keV (0.02851 Å) at ambient temperature. The ϕ range was −70° to 36° at an increment of 0.1° with an exposure time of 0.1 s per frame. The total measurement time was 106 s.

For kinematic refinement, data were first processed using the *APEX4* software package (Bruker, 2021 ▸). Frames were integrated and corrected for Lorentz and polarization effects, scan speed, background and absorption using *SAINT* (Bruker, 2021 ▸) and *SADABS* (Krause *et al*., 2015 ▸). Space-group determination based on systematic absences and *E* statistics as well as data merging was performed in *XPREP* (Sheldrick, 2008 ▸). The structure was solved by *SHELXT* (Sheldrick, 2015*a* ▸) and refined with *SHELXL* in conjunction with *ShelXle* against all data (Sheldrick, 2008 ▸; Hübschle *et al.*, 2011 ▸, Sheldrick, 2015*b* ▸). For dynamical refinement, data were processed and merged into virtual frames with *PETS2* (Palatinus, 2019 ▸). Dynamical refinement was performed with *JANA2020* (Petříček *et al.*, 2014 ▸) using the structure from kinematic refinement with fixed structural parameters. This time-efficient refinement of only ED-specific parameters, all performed within one hour, is already sufficient to clearly identify the correct enantiomorph. Further details can be found in Table 2 ▸.

**Figure 6:**
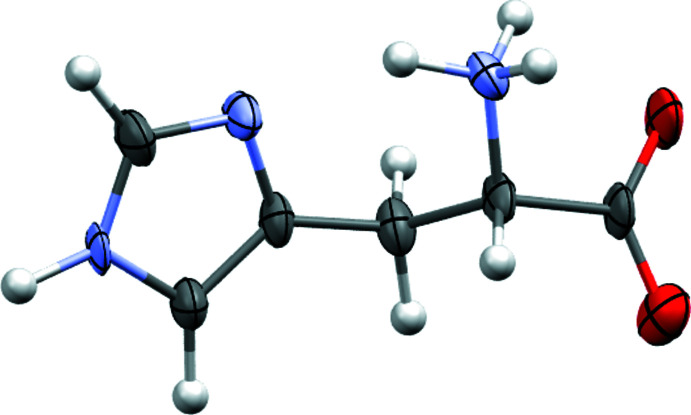
Molecular structure of l-histidine. Drawing generated using *Mercury* (Macrae *et al.*, 2020 ▸)

**Figure 7:**
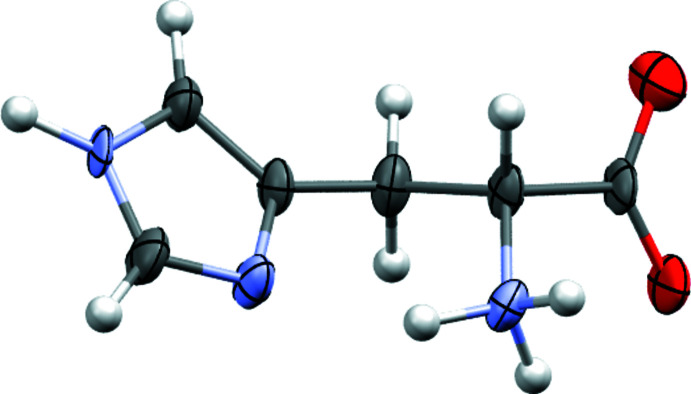
Molecular structure of d-histidine. Drawing generated using *Mercury* (Macrae *et al.*, 2020 ▸)

As shown in Table 2 ▸, the refinement for l-histidine (Fig. 6 ▸) has a lower *R*_1_ and *wR*_2_ value than the inverted structure of d-histidine (Fig. 7 ▸). By comparing the *R*-values, l-histidine can be determined as the correct enantiomer (Palatinus *et al.*, 2015*b* ▸; Brázda *et al.*, 2019 ▸).

### 1,3,5-Triphenylbenzene (TPB)

Data collected from 1,3,5-triphenylbenzene (TPB) on the Eldico ED-1 electron diffractometer at room temperature demonstrates that *R*_1_ values lower than 10% can be obtained by electron diffraction using standard kinematic refinement (Eldico Scientific AG, 2022*d* ▸).

For various reasons, there is currently still a significant discrepancy between *R*-values derived from X-ray diffraction (XRD) and electron-diffraction (ED) data. The challenges of dynamical scattering and damage of beam-sensitive samples have been discussed in Section 1.4. Furthermore, lower completeness often requires the merging of datasets from different crystals, potentially contributing to higher *R*-values in electron-diffraction structure refinements because of low-intensity correlations (Ge *et al.*, 2021 ▸). However, most crystal structures reported from electron-diffraction data were collected on instruments not optimized for ED, *i.e*. on modified transmission electron microscopes. This case study aims to capture the advantages of a dedicated electron diffractometer and its contributions to improved data quality. To focus on the improvements due to the dedicated instrumentation, TPB was selected because it is a medium-sized organic compound with low beam sensitivity and high symmetry, *i.e*. an orthorhombic space group. This compound is less prone to beam damage, and low-symmetry space groups often lead to incomplete data (Ge *et al.*, 2021*b* ▸).

Experimental conditions are summarized in Table 2 ▸. A 135° ϕ-scan was performed, cutting the high- and low-angle regions due to shading of the beam by other crystals and the TEM grid on which the sample was mounted. Data were processed and analysed using the *APEX4* (Bruker, 2021 ▸) software package and implemented programs. The frames were integrated and corrected for Lorentz effects, scan speed, background, and absorption using *SAINT* (Bruker, 2021 ▸) and *SADABS* (Krause *et al*., 2015 ▸). Space-group determination based on systematic absences and *E* statistics was performed with *XPREP* (Sheldrick, 2008 ▸). The structure was solved by *SHELXD* and refined with *SHELXL* in conjunction with *ShelXle* (Sheldrick, 2008 ▸; Hübschle *et al.*, 2011 ▸, Sheldrick, 2015*b* ▸). Non-hydrogen atoms were refined with anisotropic displacement parameters. To partially compensate for dynamical scattering and to improve the scaling and atomic displacement parameters in the kinematic refinement, an extinction parameter (EXTI) and a weighting scheme (WGHT) were used in the refinement (Wang *et al.*, 2018 ▸; Ito *et al.*, 2021 ▸). Hydrogen atoms were placed in calculated positions and refined with a riding model using inter-nuclear distances for hydrogen (Grüne *et al.*, 2014 ▸) and *U*_iso_(H) = 1.2*U*_eq_(C).

**Figure 8:**
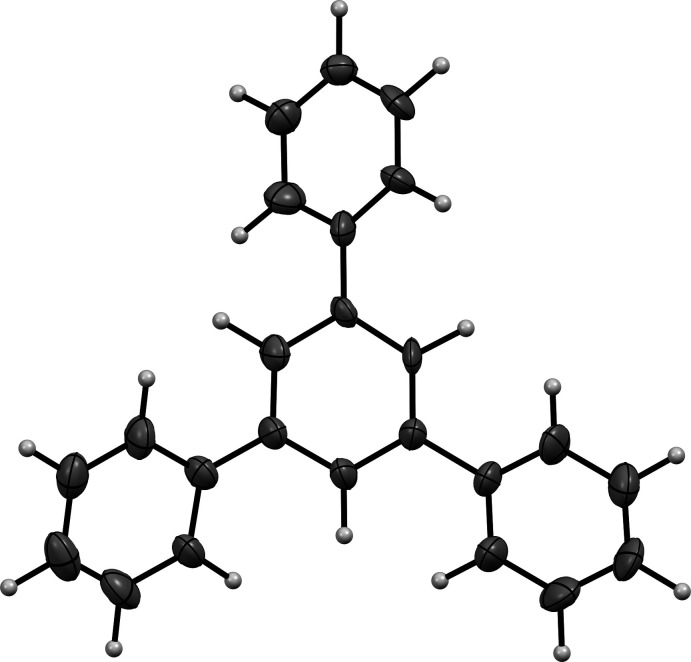
Molecular structure of TPB. Diagram generated using *Mercury* (Macrae *et al.*, 2020 ▸)

A final *R*_1_ value of 9.70% (*wR*_2_ = 28.05%) was obtained after structure refinement (Fig. 8 ▸). This *R*_1_ value below 10% is the result of standard kinematic refinement. Even without addressing the fundamental challenge of dynamical scattering in electron-diffraction experiments, dedicated electron-diffraction hardware, such as the high-precision goniometer and electron optics, and selected experimental parameters can provide data quality comparable to data collected in X-ray diffraction experiments. Crystal and refinement data are summarized in Table 2 ▸.

## Conclusion

Electron diffraction is destined to become a popular technology, enabling characterization of solid compounds in the gap between neutron, X-ray powder and single-crystal diffraction, and combining many benefits of each of these technologies. It is now feasible to examine a whole range of substances, from proteins to natural products, inorganic compounds, minerals up to advanced materials by a broad set of criteria including structure elucidation, chirality determination, examination of amorphous solid dispersions, fraud detection or polymorph screening, down to resolving information at the level of single nanometre-sized particles.

This would not have been possible without the popularization through pioneers in the field of ED, but it also required the technology to evolve to a level where detectors became sufficiently sensitive and fast on the one hand and software capable of treating effects of dynamical scattering or tracking sample movement during rotation on the other hand.

An eagerly awaited aspect is the recent introduction of the electron diffractometer, designed to be fully dedicated to its task of providing the best data from electron diffraction or scattering experiments, and at the same time able to be operated without requiring training as an electron microscopist. These criteria will contribute to the growing popularity of ED in the decades to come, and support science in its broadest sense as another powerful method for solid-state structure characterization.

## Figures and Tables

**Table 1:** The mapping operation matrix derived from the relative orientation of the two crystals

0.940	−0.094	0.088
−0.167	0.734	0.218
−1.132	−2.042	0.675

**Table 2:** Crystal information, data collection and refinement statistics

	Tyrosine		Histidine		1,3,5-Triphenylbenzene
Chemical formula	C_9_H_11_NO_3_		C_6_H_9_N_3_O_2_		C_24_H_18_
CAS number	60-18-4		71-00-1		612-71-5
Molecular weight	181.19		155.2		306.38
Crystal system	Orthorhombic		Orthorhombic		Orthorhombic
Space group	*P*2_1_2_1_2_1_		*P*2_1_2_1_2_1_		*Pna*2_1_
*a*, *b*, *c* (Å)	5.80 (6), 6.92 (6), 21.1 (2) ^*a*^		5.175 (11), 7.358 (16), 18.71 (4)		7.60 (4), 19.68 (11), 11.25 (6)
α, β, γ (°)	90, 90, 90		90, 90, 90		90, 90, 90
*V* (Å^3^)	846 (14)		712 (3)		1681 (16)
*Z*	4		4		4
Temperature	Ambient		Ambient		Ambient
Electron energy (keV)	160		160		160
Wavelength (Å)	0.02851		0.02851		0.02851
Data collection method	Single scan continuous rotation		Single scan continuous rotation		Single scan continuous rotation
ϕ range (°)	−70 to 30		−70 to 36		−70 to 65 (−47.5 to 37.5 used)
ϕ increment (°)	1		0.1		0.5
Exposure time (s/frame)	0.5		0.1		1
Total time (s)	50		106		270
Resolution (Å)	0.9		0.8		0.99
Completeness (%)	80.4^*b*^	59.9^*c*^	89.9		62.6
	86.7^*d*^				
Independent reflections	1112^*d*^		1966		1069
Parameters	122^*d*^		49		218
Restraints	246^*d*^		0		307
*R*_int_ (%)	26.26^*d*^		–^*e*^		5.60
			L-Histidine	D-Histidine	
*R*_1_ [*I* > 2σ(*I*)] (%)	14.05^*d*^		12.17^*e*^	14.01^*e*^	9.70
*wR*_2_ (all data)	37.68^*d*^		24.39^*e*^	28.65^*e*^	28.05
Goodness of fit	1.068^*d*^		4.2103^*e*^	4.9466^*e*^	1.145
					

Notes: (*a*) unit-cell parameter based on crystal 1; (*b*) completeness of data for crystal 1; (*c*) completeness of data for crystal 2; (*d*) completeness and statistics of merged data set; (*e*) statistics of dynamical refinement for L- and D-histidine.
